# Estimation of Double-Serine Phosphorylation’s Effects on the Intrinsically Disordered Region Structure in Y14 (RBM8A) Protein via Molecular Dynamics Simulation

**DOI:** 10.3390/cells15070648

**Published:** 2026-04-03

**Authors:** Yuka Nakamura, Tetsuhiro Horie, Takuya Sakamoto, Hisayoshi Yoshizaki, Hideaki Okajima, Yasuhito Ishigaki

**Affiliations:** 1 Medical Research Institute, Kanazawa Medical University, 1-1 Daigaku, Uchinada, Kahoku 920-0293, Japan; yuka-n@kanazawa-med.ac.jp (Y.N.); horie-te@kanazawa-med.ac.jp (T.H.); taku0731@kanazawa-med.ac.jp (T.S.); 2 Department of Pediatric Surgery, School of Medicine, Kanazawa Medical University, 1-1 Daigaku, Uchinada, Kahoku 920-0293, Japan; yossy@kanazawa-med.ac.jp (H.Y.); hokajima@kanazawa-med.ac.jp (H.O.)

**Keywords:** RNA binding, phosphorylation, simulation, structure

## Abstract

The C-terminus of the Y14 protein, which is also known as RBM8A and is encoded by the gene responsible for human thrombocytopenia absent radius syndrome, contains two serines that undergo phosphorylation inside an intrinsically disordered region (IDR). Although both serines are frequently phosphorylated in cells, their biological role remains unclear; therefore, we estimated the peptide structure using PEPstrMOD, which predicts peptide conformations through molecular dynamics. For this analysis, amino acid residues 151–174 of Y14, identified as an IDR in UniProt, were targeted. Structural prediction via PEPstrMOD revealed that the target peptide adopts an elongated structure in its unphosphorylated state, while simulating its phosphorylated state revealed an increase in hydrogen bonds and a more compact conformation. The compact structure of Y14 induced by phosphorylation may aid in the formation of the exon–exon junction complex at the exon–exon junction, which facilitates mRNA transport and translation. The prevalence of phosphorylated Y14 in cells may indicate that this higher-order structure is also essential for mRNA metabolism.

## 1. Introduction

Nonsense-mediated mRNA decay (NMD) is a metabolic pathway that selectively de-grades mRNAs containing a premature termination codon. It is highly conserved in eukaryotes, helping to maintain gene expression homeostasis as part of the mRNA quality control process [1,2,3]. NMD involves several factors, including the exon–exon junction complex (EJC), which forms at exon junctions of mRNA, and ribosomes [4,5,6].

Among these, the RNA-binding protein Y14, also known as RBM8A, is a core EJC component that has been reported as being the human thrombocytopenia absent radius syndrome causative gene and plays diverse roles in NMD and the cell cycle. In cells, the Y14 protein is known to form a tight heterodimer with the MAGOH protein [7,8,9,10].

Y14 contains an RNA-binding motif and two serines on its C-terminal side, Ser166 and Ser168, are phosphorylated [11]. This phosphorylation region is highly conserved across eukaryotes. Hsu et al. [11] proposed an interaction between phosphorylation and methylation in Y14, although the phosphorylation details remain unclear. Ishigaki et al. [12] re-examined phosphorylation sites using Phos-Tag analysis on serine-to-alanine Y14 mutants transiently expressed in cultured HeLa cells, showing that most Ser166 and Ser168 residues are phosphorylated in human cultured cells. Further mutation experiments revealed that phosphorylation of Ser168 was required for subsequent phosphorylation of Ser166 and both residues were nearly fully phosphorylated [12]. Kinases responsible for phosphorylating Y14 are under investigation, with GSK-3 identified as a key kinase based on comprehensive experiments, although others may also be involved [13]. In cultured human cells, these phosphorylations affect mRNA metabolism and modification and the subcellular localization of the RBM8A protein and its bound mRNA [14]. The high conservation of this region across species suggests that phosphorylation at these two sites plays an important role; however, the physiological function of RBM8A phosphorylation in vivo and its molecular mechanism remain poorly understood.

Ser166 and Ser168, known to be phosphorylated in RBM8A, are located in the serine/arginine repeat-containing region (RS region), which lacks a defined conformation and is considered an intrinsically disordered region (IDR). Its phosphorylation is known to cause structural changes and alter interactions with proteins and nucleic acids [15]. The three-dimensional structure of human Y14 has been determined via X-ray crystallography and cryo-electron microscopy, with 14 structures currently registered in the Protein Data Bank (PDB); however, none of these structures include the RS region containing Ser166 and Ser168, owing to its instability as an IDR [16,17,18].

Estimating protein conformation is important for studying protein–protein and drug interactions. However, given the required labor and equipment, in silico structural simulation is widely used. Although AlphaFold2 is a validated tool for protein structure prediction, it uses deep learning and is unsuitable for the present study as it cannot account for post-translational modifications, such as phosphorylation, given that it relies on known sequence and structure patterns [19]. A recent publication by Ramasamy et al., 2026, shows that prediction by AlphaFold3-phospho provided only modest improvement compared to using AlphaFold2 and AlphaFold3-non phospho [20]. Other tools, including I-TASSER, face similar limitations [21]. In this study, we used PEPstrMOD, which predicts the steric structure of post-translationally modified peptides, including their phosphorylated forms, using a molecular dynamics method [22]. This software can model structures of naturally or artificially modified proteins and peptides. Therefore, to estimate the effect of Ser166 and Ser168 phosphorylation on the steric structure of the C-terminal RS region in human Y14, we theoretically predicted the structure of this region (amino acid residues 151–174) using PEPstrMOD. Overall, through this study, we demonstrate that phosphorylation of Ser166 and Ser168 induces a conformational shift in the Y14 C-terminal region from an extended to a compact structure, suggesting that phosphorylation plays a structural role in regulating mRNA metabolism.

## 2. Materials and Methods

The amino acid sequence of human Y14 (encoded by the gene *RBM8A*) was obtained from UniProt: Q9Y5S9 (RBM8A_HUMAN). The region used for structure prediction was identified as an IDR via UniProt, consisting of amino acid residues 151–174 (VRGPPKGKRRGGRRRSRSPDRRRR).

PEPstrMOD (http://osddlinux.osdd.net/raghava/pepstrmod/index.php (accessed on 30 January 2023)) was used for molecular dynamics simulations [23]. First, the “Natural Peptides Module” of PEPstrMOD was used to predict the steric structure of the unphosphorylated wild type. Based on the resulting model, a phosphorylated stereostructure model was then predicted using the “PTMs of Residue Module” of PEPstrMOD. Hydrogen bonds in the conformational models were determined using Swiss-pdb Viewer [24].

The three-dimensional (3D) structure of human Y14 was visualized using Waals software version 3.0 (Altif Labs. Inc., Tokyo, Japan) referencing PDB ID: 1P27. PDB (https://www.rcsb.org), a database of protein 3D structures, was used to obtained structural data.

PEPstrMOD uses AMBER in the Natural Peptides Module and a force field library for PTM (FFPTM) compatible with AMBER in the PTMs of the Residue Module.

PEPstrMOD is made for use on a web server (https://webs.iiitd.edu.in/raghava/pepstrmod/ (accessed on10 March 2026)), so we did not set up the system. Additionally, since the analysis was conducted in a vacuum environment, solvent effects were not considered.

The parameters used for analysis are detailed below. The modules and force fields of PEPstrMOD used for structural prediction of nonphosphorylated and phosphorylated peptides, as well as the parameters of the specified options, are explained. For the former peptides, the parameters were as follows: Module: Natural Peptides; Force Field: AMBER; Simulation Time: 100 ps; and Peptide Environment: Vacuum. For the latter type of peptides, the parameters were as follows: Module: PTMs of Residue; Sub-Module: Structure Modification; SER: Phosphoserine (−2 charge); Force Field: FFPTM; Simulation Time: 100 ps; and Residues Specified: 16 and 18. In the peptide, S166 corresponded to S16 and S168 corresponded to S18.

All calculations were performed using AMBER11. The AMBER parameters used in the vacuum environment of PEPstrMOD were as follows: energy minimization, imin = 1; ntmin = 1; maxcyc = 2000; ncyc = 1000; ntb = 0; igb = 0; and cut = 10.0; production MD, imin = 0; irest = 0; ntx = 5; ntb = 0; ntc = 2; ntf = 2; cut = 10.0; igb = 0; tempi = 300; temp0 = 300; ntt = 3; gamma_ln = 2; nstlim = 100;000; dt = 0.001; ig = −1; ntpr = 100; ntwx = 100.

## 3. Results and Discussion

### 3.1. Unphosphorylated State of the Y14 C-Terminal Polypeptide Under Vacuum Environment

**Figure 1:**
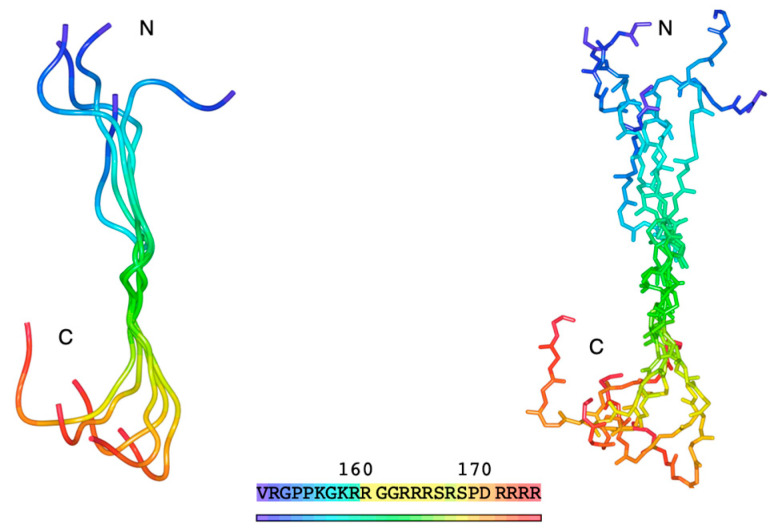
Five superimposed and predicted 3D structural models of the RS region (residues 151–174) at the Y14 C-terminus. The left panel shows the tube representation; the right panel shows the main chain structure in stick format. Colors indicate the position from the N-terminus (blue) to the C-terminus (red).

First, we predicted the theoretical conformation of the human Y14 C-terminal RS region in its unphosphorylated state using PEPstrMOD. The region modeled included the 24 residues from positions 151 to 174 (151VRGPPKGKRRGGRRRSRSPDRRRRRR174), identified as an IDR via UniProt. Conformational prediction was performed using PEPstrMOD’s “Natural Peptides Module” using the default simulation time of 100 ps. Because the targeted region is intrinsically disordered and does not adopt a stable conformation, we ran five independent simulations to explore the range of possible structures. All resulting models displayed coiled conformations lacking secondary structures, such as α-helices or β-sheets. Superimposing the five models revealed that, although the N- and C-terminal regions varied, the RS region generally adopted an extended, coiled conformation (Figure 1).

### 3.2. Y14 C-Terminal Polypeptide Phosphorylated State

**Figure 2:**
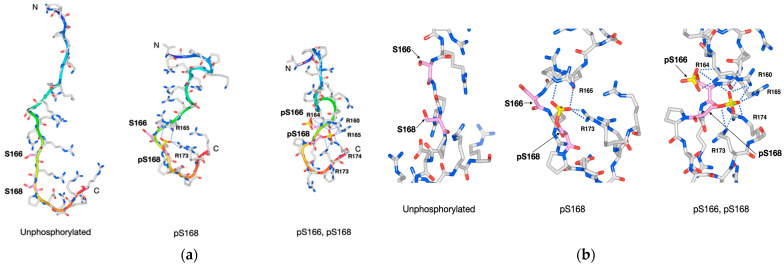
Conformational changes in the RS region at the human Y14 C-terminus upon phosphorylation. Models of the RS region in unphosphorylated, singly phosphorylated (pS168), and doubly phosphorylated (pS166 and pS168) states. (**a**) Overall peptide structures. (**b**) Enlarged views around Ser166 and Ser168. Tubes are color-graded from the N-terminus (blue) to the C-terminus (red). Amino acids are shown in stick format and atoms are color-coded: carbon (gray), oxygen (red), nitrogen (blue), and phosphorus (yellow); carbons in pSer166 and pSer168 are shown in pink. Salt bridges between phosphate groups and Arg residues are shown as blue dotted lines.

To model the phosphorylated (p) state of Ser166 and Ser168 in the Y14 C-terminal RS region, structural models were generated for the singly (pS168) and doubly phosphorylated states (pS166 and pS168) using PEPstrMOD’s “PTMs of Residue Module,” based on the five unphosphorylated models. Among the five stereostructure models (Models 1–5), that with the lowest energy value is shown in Figure 2.

In the unphosphorylated state, the peptide was relatively extended, with the side chains of Ser166 and Ser168 facing outward. In the pS168 state, the phosphate group turned inward, forming interactions with nearby arginine (Arg) residues (Arg165 and Arg173). The negatively charged phosphate group of phosphorylated serine was proposed to form a salt bridge with the positively charged side chain of a nearby Arg residue via electrostatic interaction. In the doubly phosphorylated state (pS166 and pS168), the phosphate group of pSer166 formed a salt bridge with Arg164, while that of pSer168 formed a salt bridge with Arg160, Arg164, Arg165, Arg173, and Arg174. Additionally, hydrogen bonds were predicted to exist between the phosphate groups and surrounding residues and phosphorylation-induced conformational changes were proposed to promote hydrogen bond formation between surrounding amino acid residues. Across all conformational models, the number of hydrogen bonds, including salt bridges, increased markedly in the phosphorylated compared with the unphosphorylated state (Table 1).

In Model 1 (Table 1), the number of hydrogen bonds (including salt bridges) increased from six in the unphosphorylated state to twenty-nine in the doubly phosphorylated state, an increase attributed to the conformational shifts that bring residues closer together, enabling phosphate–Arg salt bridge formation. In all five predicted conformational models, phosphorylation of Ser166 and Ser168 consistently led to the formation of salt bridges between their phosphate groups and nearby positively charged Arg or lysine (Lys) residues, along with an overall increase in hydrogen bonds within the RS region (Figure 2).

Phosphorylation made the RS region more compact relative to the original unphosphorylated state due to increased intraregional interactions and hydrogen bonding, resulting in reduced flexibility and a more stable conformation. Although comparison is meaningless due to structural differences, the theoretical energy values for the unphosphorylated RS region ranged from −1350 to −1450 kJ/mol and those for the doubly phosphorylated models were between −1550 and −1750 kJ/mol.

**Figure 3:**
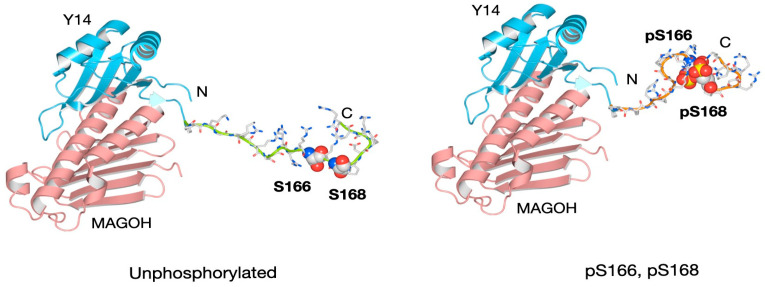
Three-dimensional structural model of human Y14 and MAGOH with Model 1 appended to the RS region of the Y14 C-terminus.

Figure 3 presents a structural model of the Y14–MAGOH complex (PDB ID: 1P27), with Model 1 appended to Y14’s C-terminus. In the unphosphorylated state, the RS region appeared elongated and flexible and was considered unstable. Upon phosphorylation, it adopted a more compact, less flexible structure and was considered stable. Arg residues that were surface-exposed in the unphosphorylated state became engaged in phosphate interactions, potentially reducing the number of residues available for binding to other proteins. These conformational changes may have influenced interactions between Y14 and its partners. As the EJC formed a compact structure on mRNA, it is plausible that phosphorylation at the two sites contributed to stabilizing the Y14–MAGOH heterodimer.

A limitation of this study is that structural estimation is performed solely through thermodynamic simulations. However, analyzing structures, especially structural changes caused by phosphorylation modifications, using crystallographic analysis methods or machine learning simulations such as AlphaFold is extremely difficult in IDR, and so further analysis using NMR or other methods will be necessary in the future.

## 4. Conclusions

In this study, we show that phosphorylation of Ser166 and Ser168 in the C-terminal RS region of Y14 likely promotes salt bridge formation between the serine phosphate groups and nearby Arg or Lys residues, along with an increase in hydrogen bonding within the RS region. Consequently, the phosphorylated state is expected to result in a more compact and stable conformation compared with the original elongated and flexible unphosphorylated form. Although the specific Arg residues interacting with the phosphate groups varied across the five conformational models, all inferred salt bridge formation between the phosphorylated serine residues and nearby Arg or Lys residues.

Similarly, in vivo, phosphate groups on phosphorylated serine residues in the flexible C-terminal RS region of Y14 may bind variably to adjacent Arg residues rather than to a fixed position. The salt bridges formed through electrostatic interactions between negatively charged phosphate groups and the positively charged side chains of Arg and Lys residues are stronger than typical hydrogen bonds and contribute to structural stabilization.

Additionally, hydrogen bonding within the RS region is expected to increase due to conformational rearrangements induced by phosphate–Arg/Lys interactions, further stabilizing the structure. The phosphorylated conformational model is consistently more compact than the unphosphorylated conformation, likely due to increased intraregional bonding, a result which aligns with previous findings from molecular dynamics simulations and small-angle X-ray scattering studies showing that phosphorylation can shrink the conformation of IDRs enriched with positively charged side chains [15].

Although this analysis is also performed in a hydrophilic environment, we are unable to adequately present a model; however, preliminary analysis results indicate that phosphorylation could adopt a folded structure similar to the vacuum environment model. Moving forward, we aim to refine our analytical methods and environment to present models incorporating interactions with water molecules, conduct full-length analyses, and investigate structural changes upon binding to other proteins.

## Figures and Tables

**Table 1:** Number of hydrogen bonds and amino acid residues forming salt bridges with phosphate groups in human Y14 C-terminus RS region conformational models.

Model	Hydrogen Bond			Salt Bridge Formation
	Unphosphorylated	pS168	pS166 and pS168	
1	6	16	29	R160, R164, R165, R173, R174
2	11	18	27	R165, R167, R172, R174
3	8	21	24	K158, R159, R167, R171, R172
4	8	20	24	R152, R164, R165, R167, R171, R173
5	12	15	21	K158, R164, R167

## Data Availability

Structural coordinate files (PDB format) obtained for Models 1 to 5 are stored at 10.6084/m9.figshare.29848436.

## Abbreviations

The following abbreviations are used in this manuscript:

IDR	Intrinsically disordered region
NMD	Nonsense-mediated mRNA decay
EJC	Exon–exon junction complex
Ser	Serine
Arg	Arginine
Lys	Lysine
RS	Serine/arginine
PDB	Protein Data Bank

## References

[1] McMahon M., Maquat L.E. (2025). Exploring the therapeutic potential of modulating nonsense-mediated mRNA decay. RNA.

[2] Patro A.K., Panigrahi G.K., Majumder S., Das R., Sahoo A. (2024). Nonsense-mediated mRNA decay: Physiological significance, mechanistic insights and future implications. Pathol. Res. Pract..

[3] Das R., Panigrahi G.K. (2025). Messenger RNA surveillance: Current understanding, regulatory mechanisms, and future implications. Mol. Biotechnol..

[4] Carrard J., Lejeune F. (2023). Nonsense-mediated mRNA decay, a simplified view of a complex mechanism. BMB Rep..

[5] Martin H., Rupkey J., Asthana S., Yoon J., Patel S., Mott J., Pei Z., Mao Y. (2022). Diverse roles of the exon junction complex factors in the cell cycle, cancer, and neurodevelopmental disorders-potential for therapeutic targeting. Int. J. Mol. Sci..

[6] Asthana S., Martin H., Rupkey J., Patel S., Yoon J., Keegan A., Mao Y. (2022). The Physiological roles of the exon junction complex in development and diseases. Cells.

[7] Ma Q., Tatsuno T., Nakamura Y., Izumi S., Tomosugi N., Ishigaki Y. (2019). Immuno-detection of mRNA-binding protein complex in human cells under transmission electron microscopy. Microsc. Res. Technol..

[8] Ma Q., Tatsuno T., Nakamura Y., Ishigaki Y. (2019). The stability of Magoh and Y14 depends on their heterodimer formation and nuclear localization. Biochem. Biophys. Res. Commun..

[9] Ishigaki Y., Nakamura Y., Tatsuno T., Hashimoto M., Shimasaki T., Iwabuchi K., Tomosugi N. (2013). Depletion of RNA-binding protein RBM8A (Y14) causes cell cycle deficiency and apoptosis in human cells. Exp. Biol. Med..

[10] Boussion S., Escande F., Jourdain A., Smol T., Brunelle P., Duhamel C., Alembik Y., Attié-Bitach T., Baujat G., Bazin A. (2020). AR syndrome: Clinical and molecular characterization of a cohort of 26 patients and description of novel noncoding variants of RBM8A. Hum. Mutat..

[11] Hsu I.W., Hsu M., Li C., Chuang T.W., Lin R.I., Tarn W.Y. (2005). Phosphorylation of Y14 modulates its interaction with proteins involved in mRNA metabolism and influences its methylation. J. Biol. Chem..

[12] Ishigaki Y., Nakamura Y., Tatsuno T., Ma S., Tomosugi N. (2015). Phosphorylation status of human RNA-binding protein 8A in cells and its inhibitory regulation by Magoh. Exp. Biol. Med..

[13] Shinde M.Y., Sidoli S., Kulej K., Mallory M.J., Radens C.M., Reicherter A.L., Myers R.L., Barash Y., Lynch K.W., Garcia B.A. (2017). Phosphoproteomics reveals that glycogen synthase kinase-3 phosphorylates multiple splicing factors and is associated with alternative splicing. J. Biol. Chem..

[14] Tatsuno T., Ishigaki Y. (2018). C-terminal short arginine/serine repeat sequence-dependent regulation of Y14 (RBM8A) localization. Sci. Rep..

[15] Modic M., Adamek M., Ule J. (2024). The impact of IDR phosphorylation on the RNA binding profiles of proteins. Trends Genet..

[16] Fribourg S., Gatfield D., Izaurralde E., Conti E. (2003). A novel mode of RBD-protein recognition in the Y14-Mago complex. Nat. Struct. Biol..

[17] Bono F., Ebert J., Lorentzen E., Conti E. (2006). The crystal structure of the exon junction complex reveals how it maintains a stable grip on mRNA. Cell.

[18] Buchwald G., Ebert J., Basquin C., Sauliere J., Jayachandran U., Bono F., Le Hir H., Conti E. (2010). Insights into the recruitment of the NMD machinery from the crystal structure of a core EJC-UPF3b complex. Proc. Natl. Acad. Sci. USA.

[19] Malhotra Y., John J., Yadav D., Sharma D., Vanshika, Rawal K., Mishra V., Chaturvedi N. (2025). Advancements in protein structure prediction: A comparative overview of AlphaFold and its derivatives. Comput. Biol. Med..

[20] Ramasamy P., Zuallaert J., Martens L., Vranken W.F. (2026). Assessing the relation between protein phosphorylation, AlphaFold3 models, and conformational variability. Protein Sci..

[21] Roy A., Kucukural A., Zhang Y. (2010). I-TASSER: A unified platform for automated protein structure and function prediction. Nat. Protoc..

[22] Singh S., Singh H., Tuknait A., Chaudhary K., Singh B., Kumaran S., Raghava G.P. (2015). PEPstrMOD: Structure prediction of peptides containing natural, non-natural and modified residues. Biol. Direct.

[23] Chauhan M., Gupta A., Tomer R., Raghava G.P.S. (2025). CancerPPD2: An updated repository of anticancer peptides and proteins. Database.

[24] Guex N., Peitsch M.C. (1997). SWISS-MODEL and the Swiss-PdbViewer: An environment for comparative protein modeling. Electrophoresis.

